# The vestibular system in pain and embodiment: cortical overlap, modulatory potential, and therapeutic perspectives

**DOI:** 10.3389/fnins.2025.1661515

**Published:** 2025-09-03

**Authors:** Nicolas Bouisset, Phivos Phylactou, Arnaud Duport

**Affiliations:** ^1^Human Threshold Research Group, Lawson Research Institute, London, ON, Canada; ^2^Vestibular Lab, Lawson Research Institute, London, ON, Canada; ^3^Department of Medical Biophysics, Schulich School of Medicine and Dentistry, University of Western Ontario, London, ON, Canada; ^4^School of Physical Therapy, Faculty of Health Sciences, University of Western Ontario, London, ON, Canada; ^5^The Gray Centre for Mobility and Activity, Parkwood Institute, London, ON, Canada

**Keywords:** vestibular stimulation, pain modulation, embodiment, multisensory integration, therapeutic perspectives

## Abstract

Musculoskeletal pain is increasingly understood as a product of disrupted multisensory integration rather than a direct consequence of tissue damage alone. Among the sensory systems involved in shaping body representation and modulating pain, the vestibular system remains largely overlooked. Beyond its classical role in balance and spatial orientation, vestibular input contributes to embodiment, self-location, and bodily self-consciousness—processes that are frequently altered in chronic pain conditions. Neuroimaging and clinical evidence reveal a striking overlap between vestibular integration regions and the so-called pain neuromatrix, suggesting shared cortical substrates for vestibular and nociceptive/pain processing. Moreover, vestibular dysfunction is associated with disembodiment phenomena such as depersonalization and derealization, which mirror sensory distortions observed in chronic pain syndromes. Experimental studies demonstrate that vestibular stimulation—via caloric or electric modalities—can modulate pain perception, influence somatosensory integration, and recalibrate distorted body representations. This perspective paper synthesizes current findings at the intersection of vestibular neuroscience, pain modulation, and embodiment, proposing that the vestibular system could constitute a critical but underrecognized component in musculoskeletal health. Incorporating vestibular pathways into pain models may, therefore, improve our understanding of chronicity and open novel therapeutic avenues for neuromodulation.

## Introduction

Pain in musculoskeletal disorders is not solely a reflection of tissue injury but often emerges from complex interactions between sensory input, cognitive appraisal, and body representation, especially when it comes to chronic pain (Wang and Frey-law, 2024). Contemporary pain neuroscience emphasizes the brain’s central role in shaping pain perception and modulating somatic experience through multisensory integration (Wang and Frey-law, 2024). One particularly underexplored but potentially crucial system in this integrative network is the vestibular system.

Beyond its classical role in balance and spatial orientation (Cullen, 2019), the vestibular system contributes to higher-order processes including self-location, bodily self-consciousness, and embodiment (Hitier et al., 2014; Mast et al., 2014). These functions are mediated through multisensory interactions in key cortical areas such as the temporoparietal junction (TPJ), insula, and posterior parietal cortex (Lopez and Blanke, 2011)—regions that also play a pivotal role in pain processing (Wang and Frey-law, 2024). The overlap between vestibular integration centers and the so-called “pain neuromatrix” (Moseley, 2003) suggests a potential modulatory role of vestibular input on pain perception and embodiment.

Vestibular dysfunction has been associated with depersonalization, derealization, and altered body schema (Kolev et al., 2014; Jáuregui Renaud, 2015; Elyoseph et al., 2023), which are phenomena that bear striking resemblance to the sensory distortions often reported in chronic pain states (Wang and Frey-law, 2024). Furthermore, experimental evidence demonstrates that vestibular stimulations such as caloric stimulation or galvanic vestibular stimulation (GVS; also recently referred to as electric vestibular stimulation- EVS) can modulate pain perception (André et al., 2001; Le Chapelain et al., 2001; Ramachandran et al., 2007b; McGeoch and Ramachandran, 2008; McGeoch et al., 2008; Ferrè et al., 2015b; Spitoni et al., 2016; Wilkinson et al., 2017), modulate tactile thresholds (Ferrè et al., 2011, 2012, 2013, 2014), and even restore altered body representations in both healthy individuals and clinical populations (André et al., 2001; Le Chapelain et al., 2001; Rode et al., 2012).

In this perspective paper, we explore the intersection of vestibular neuroscience, pain modulation, and embodiment mechanisms. We propose that the vestibular system constitutes a missing link in our understanding of pain and bodily disintegration in musculoskeletal disorders. By integrating insights from neurophysiology, cognitive neuroscience, and clinical research, we aim to open new perspectives on how vestibular inputs can influence and potentially alleviate pain and body schema disruptions in musculoskeletal health.

## Vestibular system and body representation

The transient modulation of body representation related to the body schema can rapidly be achieved through visuo–proprioceptive integration (Blanke, 2012). In the Pinocchio illusion for instance, as their vision is obstructed, individuals perceive their own nose as growing longer when the tendons of their biceps are vibrated. Conversely, when vibrations target the triceps, then participants feel their nose being pushed inside their heads, underlining a need for the brain to make sense of incongruous information (Lackner, 1988). Another commonly used paradigm for self-consciousness and body ownership is the so called rubber hand illusion (Botvinick and Cohen, 1998). In this case, participants perceive a fake hand as their own when they see it being brushed in sync with their hidden real hand. Moreover, embodiment illusions do not limit themselves to some specific body parts, as entire body illusions can be elicited as well. Indeed, following the same temporal, spatial and anatomical constraints (Ehrsson, 2012), illusions such as “full body” (Petkova et al., 2011), “out of body” (Ehrsson, 2007; Lenggenhager et al., 2007), “swapping bodies” (Petkova and Ehrsson, 2008) can also be produced. These paradigms provide evidence that brain’s involvement in body representation and self-consciousness is very plastic and can be easily modified through sensory integration processes.

Interestingly, vestibular patients often report depersonalization and derealization symptoms (Kolev et al., 2014; Jáuregui Renaud, 2015; Elyoseph et al., 2023). Depersonalization is described by feelings of unreality, detachment, or the sensation of being an external observer when it comes to one’s thoughts, emotions, physical sensations, or actions. Derealization, on the other hand, relates to feelings of unreality or detachment concerning one’s surrounding (Guze, 1995). Such vestibular patients describe experiences such as “not being in control of self” or reporting “their body feeling strange” (Smith and Darlington, 2013), suggesting feelings of disembodiment when vestibular dysfunction occurs (Lopez et al., 2008). Furthermore, although quite rare, neurological patients with lesion sites found where vestibular inputs are highly integrated, such as at the right TPJ, can experience out-of-body experiences (Blanke et al., 2004; Lopez and Elzière, 2018). Indeed, self-location and the first-person perspective rely on the integration of visual and somatic inputs along vestibular signals (Blanke, 2012), suggesting the vestibular information helps in anchoring the visuo-spatial perspective to the body (Pavlidou et al., 2018). Moreover, further supporting this view, a rare case report demonstrated that direct subcortical stimulation of the left TPJ during awake craniotomy elicited reproducible out-of-body experiences (Bos et al., 2016). This case illustrates that disrupting vestibulo-cortical processing alone can transiently alter self-location, reinforcing the notion that the vestibular system is fundamentally involved in the neural mechanisms underlying embodiment and bodily self-awareness.

Additionally, vestibular-specific stimulations modulate body schema and size perception in both patients and healthy individuals (Lopez et al., 2018). For instance, such stimulations can modify the shape as well as the spatial orientation of phantom limbs (André et al., 2001; Le Chapelain et al., 2001), temporally alleviate enlarged and distorted face perception (Rode et al., 2012), restore body misrepresentation in patients with somatoparaphrenia (Spitoni et al., 2016) and improvement in hemi-spatial neglect (Karnath and Dieterich, 2006; Sturt and Punt, 2013). In healthy participants, despite contrasting results, vestibular stimulations such as GVS modulate the effect of the rubber hand illusion (Lopez et al., 2010; Ferrè et al., 2015a; Ponzo et al., 2018). Furthermore, vestibular stimulations also modify shape and size of healthy limbs. This is all the more important, knowing that distorted body perceptions are often linked to pain (Boesch et al., 2016), as discussed next.

**Figure 1:**
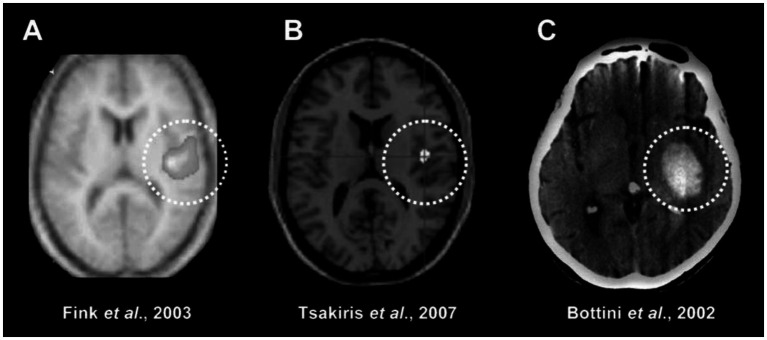
Some overlapping brain regions for vestibular processing and body parts ownership. **(A)** Left anodal GVS/right cathodal GVS (excitation of the right and inhibition of the left vestibular apparatus) induces a significant BOLD signal increase in the right posterior insula, superior temporal gyrus and anterior inferior parietal cortex. After **(B)** In a positron emission tomography study, ownership of a fake hand (proprioceptive drift toward the rubber hand) was positively correlated to BOLD signal in the right posterior insula. After **(C)** A 77 year-old right-handed woman suffering from somatoparaphrenia for her left hand (which she attributed to her niece) had a hemorrhagic lesion involving the white matter underlying the right insula, superior temporal gyrus, parietal operculum, and the precentral and postcentral gyri. After Figure and caption reprinted from Lopez et al. (2010), Copyright (2010), with permission from Elsevier.

Neuroimaging data reveal that crucial brain regions engaged in vestibular processing overlap with areas associated with multisensory integration and mechanisms related to embodiment (Ehrsson et al., 2004; Tsakiris et al., 2008; Olivé et al., 2015; Ehrsson, 2019). The primary cortical convergence for these processes predominantly occurs at the TPJ (Figures 1A–C), encompassing the posterior insula, posterior parietal cortex, and premotor cortex. Additionally, insights into the involvement of the right TPJ and posterior insula in the sense of body ownership are gained from studies involving neurological patients with abnormal ownership senses, such as Somatoparaphrenia (Figure 1C). In addition to the right TPJ, posterior parietal cortex and posterior insula, a growing body of work identifies area OP2 in the parietal operculum as the central hub of the human vestibular cortex. Meta-analytic, task-based and connectivity studies show that OP2 is the only cortical site consistently activated by all forms of vestibular stimulation, displays vestibular-specific responses dissociable from other input, and possesses sub-regional networks that integrate vestibular, somatosensory and visual information while predicting both healthy and pathological states (Zu Eulenburg et al., 2012; Raiser et al., 2020; Huber et al., 2021; Ibitoye et al., 2023).

**Figure 2:**
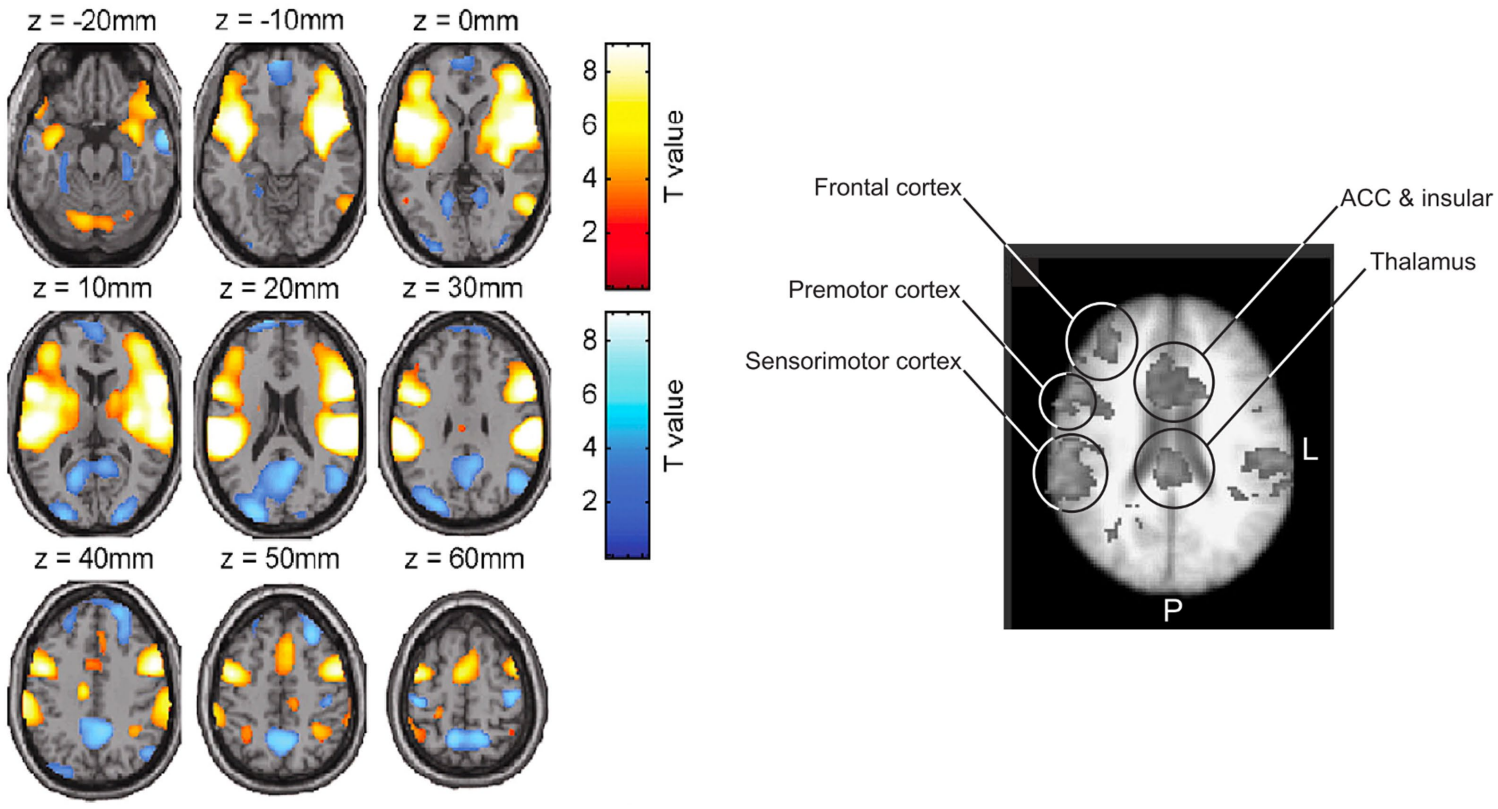
Cortical overlap between vestibular and pain integration regions. Left panel: Brain activation (yellow/red) and deactivation (blue) maps during galvanic vestibular stimulation (GVS), across all stimulation frequencies. Activation clusters include the supramarginal gyrus, lateral sulcus, superior temporal gyrus, anterior and posterior insula, inferior/middle frontal gyri, anterior cingulate cortex, and precentral sulcus. Deactivations appear in bilateral precuneus, precentral gyrus, middle occipital and temporal gyri, parahippocampal regions, and medial/superior frontal areas. Figure reprinted from Stephan et al. (2005), Copyright (2005), with permission from Elsevier. Right panel: fMRI activation during thermal pain stimulation, illustrating core regions of the pain neuromatrix: thalamus, anterior cingulate and insular cortices, frontal, premotor, and sensorimotor areas. Figure reprinted from Moseley (2003), Copyright (2003). Reproduced with permission from Elsevier.

**Figure 3:**
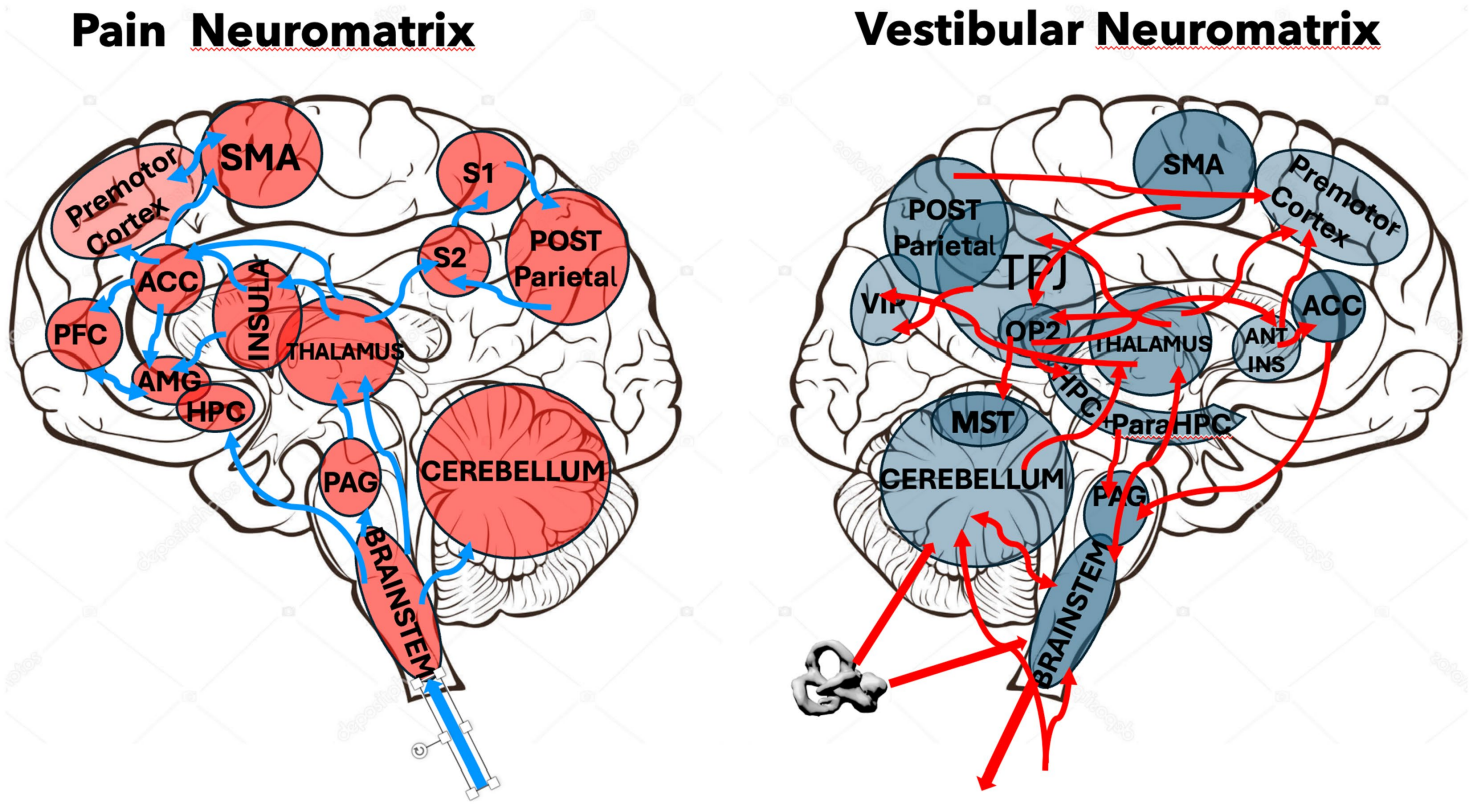
Schematic comparison of simplified pain and vestibular neuromatrices. The left panel depicts the principal cortical, subcortical, and brainstem regions comprising the pain neuromatrix (red), while the right panel illustrates the major components of the vestibular neuromatrix (blue). Areas and pathways shared by both networks are readily identifiable, including the anterior cingulate cortex (ACC), posterior parietal cortex, supplementary motor area (SMA), premotor cortex, thalamus, periaqueductal gray (PAG), cerebellum, and brainstem nuclei. Arrows indicate major connections between nodes within each network. This schematic is a simplified representation designed to emphasize key anatomical similarities and shared connectivity patterns, rather than an exhaustive depiction of all known projections.

Therefore, by examining the literature on (1) cortical integration during vestibular stimulations (Figure 1A), (2) brain activity in embodiment experiments (Figure 1B), and (3) post-stroke imaging in patients with body schema impairments (Figure 1C), it can be inferred that there is a strong overlap of brain regions between vestibular processing and body ownership. Considering the above, the vestibular system seems to be decisively implicated in embodiment mechanisms and increasing data link vestibular integration to body schema construction (Lopez and Blanke, 2007; Schwabe and Blanke, 2008; Lopez et al., 2010, 2012b; Blanke, 2012; Lopez, 2013, 2016; Mast et al., 2014; Peiffer et al., 2014; Lenggenhager and Lopez, 2015).

## Pain modulation via vestibular pathways

Just like for the vestibular system, it is fascinating to see that neuroanatomical investigations reveal the absence of a single cortical area dedicated to pain. As there is no single vestibular integration center (Lobel et al., 1998; Bense et al., 2001; Stephan et al., 2005; Lopez and Blanke, 2011) (Figure 2, left panel), there is no “pain center” within the human brain (Figure 2, right panel). Indeed, many brain areas are implicated in the emergence of pain and it is worth noting that an important activation variability exists between and within individuals depending on pain states and perception (Crawford et al., 2023). That being said and acknowledged, some brain areas seem more often involved than others and represent a cerebral core network referred to as the “pain neuromatrix” (Moseley, 2003) (Figure 2, right panel), in reference to Melzack’s ‘Neuromatrix theory’ (Melzack, 1990, 1996). As reported by Moseley (2003) the thalamus, the anterior cingulate cortex (ACC), but also insular, frontal, premotor and primary sensory and motor, as well as the posterior parietal cortices are the principal brain components of the pain neuromatrix (Figure 2, right panel).

Interestingly, all regions found within the pain neuromatrix are also largely modulated with vestibular-specific stimulations (Lobel et al., 1998; Bense et al., 2001; Stephan et al., 2005; Lopez and Blanke, 2011; Lopez et al., 2012a; Hitier et al., 2014; Habig et al., 2023) (Figure 2, left panel), implicating important cortical overlap and shared information processing within multisensory integration centers (Balaban, 2011).

Moreover, ultra-high-field imaging now delineates a coherent vestibulo-autonomic-nociceptive circuit with a vestibular-only cortical core. Resting-state 7 T fMRI places the vestibular nuclei (Ve) in strongest functional coupling with thalamus, parietal operculum OP2 and posterior insula, while second-order links reach a brain-stem autonomic–nociceptive cluster that includes the lateral/medial parabrachial nuclei, medullary reticular formations and periaqueductal gray; connectivity to the raphe complex is minimal (Cauzzo et al., 2022). Parallel 7 T diffusion-tractography uncovers an almost mirror-symmetric structural scaffold: Ve fibers course through the inferior olive and fastigial/lobule X, then ascend to thalamus, insula and cingulate, and extend to the parabrachial–PAG axis, again sparing raphe projections (Singh et al., 2022). Critically, task fMRI that directly contrasts galvanic vestibular with equally salient nociceptive stimulation confirms OP2 as a vestibular-selective node, whereas OP1/3/4 and anterior insula respond preferentially to nociception; only the nociceptive condition reorganizes whole-brain functional networks, underscoring the continuous, background nature of vestibular processing. A recent systematic review of pain imaging adds that cerebellar lobules IV–VI and Crus I—regions receiving monosynaptic input from Ve and fastigial nuclei—integrate sensorimotor, affective and cognitive dimensions of pain (Li et al., 2024) underlying a “‘mysterious” cerebellar role in pain modulation. Together, these converging functional, structural and task-based data trace a pathway that is vestibular-specific at OP2, but merges with autonomic and nociceptive systems downstream, providing a mechanistic framework for the frequent co-occurrence of dizziness, anxiety and pain and suggesting testable circuit-level targets for neuromodulatory therapy (Figure 3).

Thus, given that impressive vestibulo-autonomic-nociceptive network (Cauzzo et al., 2022; Singh et al., 2022; Li et al., 2024) but also the large overlap between the pain neuromatrix, embodiment networks, and the vestibular cortical regions (Figure 3), stimulating the vestibular system could help with relieving pain by gate controlling or “masking pain processing” as hypothesized by Ramachandran et al. (2007b).

Indeed, as hypothesized by Harris (1999), the literature reports that strong vestibular stimulations such as caloric stimulation can relieve pain (André et al., 2001; Le Chapelain et al., 2001; Ramachandran et al., 2007b; McGeoch and Ramachandran, 2008; McGeoch et al., 2008; Ferrè et al., 2015b; Spitoni et al., 2016; Wilkinson et al., 2017). Moreover, such vestibular stimulations help in relieving central poststroke pain (Ramachandran et al., 2007a; McGeoch et al., 2008) considered by some as the “*most distressing, and intractable of pain syndromes* “which normally are “*largely refractory to medical and surgical treatments*” (Henry et al., 2008). In these central pain states, caloric stimulation is thought to modulate multisensory cortical areas such as the posterior insula and parietal cortex involved in both nociceptive perception and vestibular integration (Ramachandran et al., 2007a, 2007b; McGeoch and Ramachandran, 2008; McGeoch et al., 2008; Naryshkin et al., 2023). Other mechanisms have been hypothesized where other parts of the brain such as the ACC would be modulated to inhibit the pain perception (McGeoch and Ramachandran, 2008; Spitoni et al., 2016). Besides patients, caloric vestibular stimulations also inhibit laser-induced experimental nociceptive inputs in healthy participants (Ferrè et al., 2015b).

Electric vestibular-specific stimulations are also known to activate the insular cortex (Bucher et al., 1998; Lobel et al., 1998; Bense et al., 2001; Stephan et al., 2005), which could potentially initiate anti-nociceptive effects through its physiological action on insular nociceptive networks.

## Vestibular influence on somatosensory integration

Pain is often also uncorrelated with the actual state of the tissues (Moseley and Vlaeyen, 2015). Pain also emerges, most of the time, as a brain response to perceived bodily danger (Moseley and Flor, 2012), pushing one to seek a solution to real or potential harm.

In the case of musculoskeletal pain, the way proprioception is integrated is often altered (Hänsel et al., 2011). This condition is frequently associated with reduced proprioceptive acuity and diminished bodily awareness (Tong et al., 2017), forcing the brain to, in some instances, reweight the proprioceptive gains between different body parts (Brumagne et al., 2004; Goossens et al., 2019). Altered proprioceptive inputs can modulate integrative processes inducing plastic changes both at the dorsal horn of the spinal cord and at the cortical level (Brumagne et al., 2019) potentially causing cortical maps reorganizations responsible for lingering pain inadaptations (Tsao et al., 2008, 2011; Moseley and Vlaeyen, 2015; Schabrun et al., 2015, 2016, 2017).

The secondary somatosensory cortex as well as the insula and the retroinsular cortex all receive vestibular inputs (Bottini et al., 2001; Lopez and Blanke, 2011; Lopez et al., 2012a). Thus, there’s overt overlap between tactile, proprioceptive, and vestibular cortical maps providing a neurophysiological explanation for vestibular influence on somatic inputs. Indeed, vestibular stimulations have been found to enhance or restore subtle somatosensory stimuli awareness in both healthy participants (Ferrè et al., 2011, 2012, 2013, 2014) and neurological patients (Vallar et al., 1990, 1993; Bottini et al., 1995; Kerkhoff et al., 2011; Schmidt et al., 2013).

Therefore, these studies underline the importance of vestibular-somatic interactions. Thus, vestibular inputs could be useful in reweighting proprioceptive inputs and helping with somatic acuity and awareness which seems to be impaired due to musculoskeletal dysfunction and pain. By enhancing the integration of proprioceptive and tactile inputs, engaging the vestibular system may help preserve the topographic specificity of cortical sensorimotor representations. This is particularly relevant for preventing “smudging”—a phenomenon marked by increased overlap between cortical representations of adjacent body parts (Tsao et al., 2011; Schabrun et al., 2017). Through its role in promoting adaptive plasticity and multisensory integration, vestibular stimulation could therefore reduce the risk of maladaptive reorganization and help prevent the transition from acute to chronic pain states (Senkowski and Heinz, 2016).

## Therapeutic perspectives

The current literature provides a strong theoretical backbone supporting that vestibular stimulation could be a potent approach for modulating embodiment and pain mechanisms. Thus, the rehabilitation process might benefit from utilizing tools such as Caloric vestibular stimulations or GVS. For instance, vestibular stimulation might serve as a useful tool to help enhance the integration of somatosensory cues. Moreover, GVS was reported to enhance visual capture and modulate proprioceptive cues during rubber hand experiments (Lopez et al., 2010). A growing pool of pain modulation techniques capitalize on multisensory integration, particularly through visuo-proprioceptive and visuo-tactile channels, to recalibrate distorted body representations and reduce pain. Notable examples include mirror box therapy (Ezendam et al., 2009), which uses mirrored visual feedback to resolve sensorimotor incongruence in phantom limb pain (Chan et al., 2007) or Complex Regional Pain Syndrome (McCabe et al., 2003), graded motor imagery (Bowering et al., 2013), which progresses through imagined movement and mirror therapy to normalize cortical excitability, and immersive virtual reality paradigms (Li et al., 2011) that re-anchor bodily self-consciousness through first-person visual feedback. Devices like the “Mirage” box (Newport and Gilpin, 2011; Preston and Newport, 2011; Gilpin et al., 2015) further exploit dynamic visual distortions to modulate body size perception and pain intensity (Preston and Newport, 2011; MacIntyre et al., 2019). These approaches are all based on the premise that modifying how the body is visually and proprioceptively experienced can influence cortical representations and, by extension, nociceptive processing. Despite their promise, such interventions may not fully address deeper multisensory disintegration—especially when vestibular input, a key contributor to self-location and embodiment, is disregarded. Integrating vestibular stimulations alongside these therapies could reinforce their effects by stabilizing body schema and enhancing central coherence across sensory modalities. For example, applying GVS during mirror therapy might enhance proprioceptive anchoring and reduce conflicting sensory signals, potentially yielding greater pain relief and embodiment restoration. Similarly, combining GVS with VR-based interventions could augment presence and agency by engaging vestibulo-cortical circuits critical for self-location and bodily awareness. Thus, vestibular input may serve as a neuromodulatory scaffold, priming the nervous system for more effective integration of visual, tactile, and proprioceptive cues.

While movement-based vestibular stimulation could also hold therapeutic potential, our particular focus on vestibular-specific stimulations herein stems from the need to first establish a mechanistically precise and experimentally controlled link between vestibular input and embodiment and pain modulation. GVS for instance provides a well-characterized method to selectively activate the vestibular system without engaging concurrent motor or proprioceptive systems, allowing us to isolate vestibular contributions and implement robust sham-controlled designs. This level of experimental control is crucial at this first stage, where the primary objective would be to demonstrate a more causal interaction. That said, the insights gained from GVS-based paradigms could provide a foundational framework for the development of movement-based vestibular interventions. Once the underlying mechanisms are clarified, natural stimulation approaches could indeed represent a more accessible and ecologically valid means of harnessing vestibular pathways for pain modulation in clinical populations.

Besides what we have already covered, the vestibular system is also linked with autonomic system functions (Yates and Bronstein, 2005; Yates et al., 2015; Rajagopalan et al., 2017). It has been shown to impact autonomic reflexes such as the vestibulo-sympathetic reflex (Samoudi et al., 2012) modulating blood pressure, heart rate, and cerebral blood flow (Yamamoto et al., 2005; Cohen et al., 2013; Yakushin et al., 2014; Yates et al., 2015). Furthermore, the otolithic system is known to play a major role in regulating circadian rhythms, homeostasis and body composition possibly due to vestibulo-hypothalamic connections (Fuller et al., 2002). Also, through limbic system connections (Balaban, 2004), the vestibular system plays a role in regulating emotions, affective processes and disorders (Mast et al., 2014; Miller, 2016; Rajagopalan et al., 2017) such as anxiety (Balaban and Thayer, 2001), and mood (Winter et al., 2012, 2013). Finally, the vestibular system is also related to sleep (Besnard et al., 2018) which is often disturbed by musculoskeletal pain (Keeffe and Fullen, 2011). All the above-mentioned points are important parameters to consider when treating musculoskeletal problems and should therefore be further investigated.

Despite the growing body of evidence linking vestibular stimulation to pain modulation and embodiment, key translational steps remain missing. Most notably, systematic assessments of vestibular function in chronic musculoskeletal pain populations are lacking. It remains unclear whether subtle vestibular deficits—perhaps subclinical—are present in these patients and contribute to sensory disintegration or distorted body representations. Identifying such deficits could help stratify patients who might benefit most from vestibular-based interventions. Future studies should include standardized vestibular testing. Tests such as Vestibular Evoked myogenic Potentials (e.g., OVEMPs, CVEMPs; Rosengren et al., 2010), video Head Impulse tests (MacDougall et al., 2009; Alhabib and Saliba, 2017; Halmagyi et al., 2017) or perceptual thresholds (Rey et al., 2016; Kobel et al., 2021) could be used in pain cohorts to determine whether vestibular dysfunction is a contributing factor or therapeutic target. Additionally, combining vestibular stimulation with current multisensory therapies in controlled trials will be critical to establish causal efficacy and guide clinical adoption. These steps are essential for moving beyond theoretical plausibility toward potential personalized, vestibular-informed rehabilitation approaches.

## Conclusion

The vestibular system, long considered primarily a mediator of balance and spatial orientation, is emerging as a pivotal contributor to higher-order bodily functions such as embodiment and pain modulation. Growing evidence suggests that vestibular inputs influence body representation, somatosensory integration, and emotional experience—domains that are profoundly altered in chronic pain conditions. The overlap between vestibular integration and pain-related cortical networks points to a potential powerful, yet underrecognized, modulatory role of the vestibular system in musculoskeletal health. Incorporating vestibular pathways into the conceptual framework of pain neuroscience not only has the potential to deepen our understanding of pain chronification but also opens new therapeutic avenues. Vestibular neuromodulation, through caloric or electric stimulations may offer a novel adjunct strategy for restoring sensorimotor coherence and alleviating pain. Future research should aim to further elucidate the mechanisms by which vestibular signals interact with the pain matrix and assess the clinical efficacy of vestibular-based interventions in chronic pain syndromes.

## Data Availability

The original contributions presented in the study are included in the article/supplementary material, further inquiries can be directed to the corresponding author.
